# Outlier Vehicle Trajectory Detection Using Deep Autoencoders in Santiago, Chile

**DOI:** 10.3390/s23031440

**Published:** 2023-01-28

**Authors:** Billy Peralta, Richard Soria, Orietta Nicolis, Fabrizio Ruggeri, Luis Caro, Andrés Bronfman

**Affiliations:** 1 Facultad de Ingeniería, Universidad Andres Bello, Av. Antonio Varas 880, Santiago 7500971, Chile; 2 Institute of Applied Mathematics and Information Technologies, National Research Council (IMATI-CNR), 20133 Milano, Italy; 3 Departamento de Ingeniería Informática, Universidad Católica de Temuco, Temuco 4781312, Chile

**Keywords:** outlier detection, GPS, vehicle trajectory, deep learning

## Abstract

In the last decade, a large amount of data from vehicle location sensors has been generated due to the massification of GPS systems to track them. This is because these sensors usually include multiple variables such as position, speed, angular position of the vehicle, etc., and, furthermore, they are also usually recorded in very short time intervals. On the other hand, routes are often generated so that they do not correspond to reality, due to artifacts such as buildings, bridges, or sensor failures and where, due to the large amount of data, visual analysis of human expert is unable to detect genuinely anomalous routes. The presence of such abnormalities can lead to faulty sensors being detected which may allow sensor replacement to reliably track the vehicle. However, given the reliability of the available sensors, there are very few examples of such anomalies, which can make it difficult to apply supervised learning techniques. In this work we propose the use of unsupervised deep neural network models based on stacked autoencoders to detect anomalous routes in vehicles within Santiago de Chile. The results show that the proposed model is capable of effectively detecting anomalous paths in real data considering validation given by an expert user, reaching a performance of 82.1% on average. As future work, we propose to incorporate the use of Long Short-Term Memory (LSTM) and attention-based networks in order to improve the detection of anomalous trajectories.

## 1. Introduction

Currently there is a large amount of data generated by the massification of various information systems, where many of them are based on sensors [1]. These data typically need to be channelled and processed in order to be transformed into useful information for the people involved in data analysis. In general, they are enormous and also poorly structured, making difficult their manual analysis. A recent alternative to deal with them is to use the machine learning methods, which consist of learning to automatically recognise patterns from data [2].

In particular, the sensors are applied in the vehicle telemetry sector in cities, which provide large volumes of data generated by the continuous monitoring of vehicles through installed GPS devices [3]. During a measurement, each vehicle sends its temporal and spatial information, as well as sensor values, where such data are normally stored in the cloud. Vehicle monitoring allows companies to make better routing decisions for drivers and detect slow traffic zones; from a governance point of view, this information can allow the creation of localized traffic policies [4]. Moreover, the detection of outlier trajectories monitored by the authorities can be important for people’s urban safety [5].

In the context of vehicle telemetry considering GPS sensors, there is the possibility that there are errors in the GPS measurements provided by the satellites. The sources of ranging/positioning errors of GPS sensors in cities are mainly due to atmospheric interference caused by possible signal delays in the ionosphere, to correction and rounding errors caused by spatial events affecting the correction models based on altered GPS signals, to satellite data errors due to changes in the position of the satellites in relation to the measurement time, and to multi-path errors caused by signal reflection from surfaces close to the receiver, which is a common event in urban environments (see, for example, [6,7,8,9], among others). These errors may generate anomalous data.

In terms of GPS sensor data, three types of anomalies are described in [10]: distance-based, velocity-based, and acceleration-based. Each of them refers to abrupt changes in patterns in each of the measures considered. Note that all these anomalies can be caused by errors in the ranging or GPS positioning. On the other hand, these anomalies can also be caused by the loss of the GPS signal due to various factors in the urban context such as entering a tunnel, proximity to buildings, or the malfunction of the GPS device itself [11], which can be reflected in abrupt measurement changes. Note that manual labeling of these anomalies can be tedious due to the noisy nature of GPS data.

Detection of sudden and sharp increasing errors can be performed by means of models based on minimum and maximum velocity constraints. However this scheme may fail to detect errors where speed errors cause out of bounds values such as one meter deviations from the value measured by the GPS [12]. Furthermore, by adding the sequence of measurements, the errors can accumulate, increasing the total error of the GPS device [13].

Detection of slowly increasing positioning errors may therefore be less obvious to perform than abrupt errors. An alternative is to use models based on space-time graphs that consider the geometrical and topological characteristics of the streets as well as the temporary restrictions on vehicle speeds [14]. Unfortunately this method requires several steps as well as non-trivial parameters. There are many other approaches that can be applied to detect this type of error, like those based on statistical [15], logical [16], data outliers [17] and clustering methods [18].

Although there is a wide variety of applicable methods [12], they usually consider several non-trivial parameters that may not necessarily lead to capturing the non-linear patterns of the sequence of GPS measurements since they are based on linear statistical assumptions. Therefore, an alternative is the use of deep neural networks for the detection of outliers in GPS trajectories, since they are models that allow the capture of the non-linear and complex relationships between the variables measured by GPS devices.

In particular, the identification of anomalous values in GPS trajectories can use methods for the detection of time series outliers, since GPS normally provides measurements at regular intervals over time. In the last decade, many methods have been proposed in various fields to detect anomalies in time series.

Yan et al. [19] propose an anomaly detection design with the unsupervised learning approach for multivariate observations, using a *stacked denoising autoencoders* (SDAE) network, which learns more robust features for the input noise. The learned features of the SDAE are taken as input to an extreme learning machine (ELM) to classify whether an observation is anomalous or not. An ELM has connections between input and hidden neurons that are randomly generated and arranged. Singh [20] proposes an unsupervised approach for univariate time series anomaly detection by combining a long- and short-term memory (LSTM) network and a recurrent neural network (RNN). The LSTM-RNN model is trained only with normal data and without anomalies; in this way the model learns the normal behavior of the time series; thus when the model is used for predictions of new data, it will give a higher prediction error in regions with anomalies compared to normal regions. An outlier detection method for univariate observations is also proposed by Ma et al. [21]. In [21], the time series is divided into subsequences using a sliding window, then an autoregressive (AR) prediction model is applied to each subsequence to predict the value of the next point. Finally, they calculate a prediction confidence interval (PCI) based on the nearest neighbor’s historical data, where, if the predicted value falls outside the PCI, it will be considered an outlier value. Lu et al. [22] face the issue of the detection of multivariate outlier time series with an unsupervised approach using deep learning. Their model integrates a denoising autoencoder network (DAE) that is used to automatically extract features from raw data and a recurrent network (RNN) which is used to model the temporal structure. The goal of the DAE network is to capture the intrinsic difference in densities between outlier and normal instances, while the RNN network seeks to obtain contextual information and, therefore, improve the construction of the features. For the identification of outliers they use the reconstruction error so that, if the time series has a high reconstruction error, then it is considered an outlier. Cao et al. [23] introduce a neighborhood-based time stream outlier detection algorithm. This method is based on the estimation of the neighborhood of trajectories in a database considering the estimation of an outlier factor, which is based on the number of data outliers in the trajectory. On the other hand, outlier data are based on the distance of each point from its neighborhoods.To speed up the calculations consider a data tree representation. Eldawy et al. [24] present the FraudMove model for online detection of abnormal trajectories in taxis. In this work, anomalies are detected by comparing the time traveled with respect to the estimated time according to the proposed model. The time estimate is based on the application of the Viterbi algorithm to infer the most probable route according to the route followed at the moment, and then dynamically estimate the time needed to reach the route.

Recently, Han et al. [25] propose DeepTEA (deep-probabilistic-based time-dependent anomaly detection algorithm) which is a hybrid model of neural networks and probabilistic models for detecting anomalous trajectories. In this work, the average speeds of vehicles are spatially represented on grids to model the traffic condition at each time, then a convolutional network is used at the spatial level, and later a LSTM network at the temporal level. This method allows to obtain time-dependent outliers from a huge volume of trajectories and detect them accurately.

In the context of GPS detection anomalies, Kieu et al. [26] present two models for the detection of anomalous time series for multivariate observations using an autoencoder-based convolutional neural network (2DCNN-AE) and an autoencoder-based Long Short Term Memory network (LSTM-AE). Furthermore, they propose a method to enrich multidimensional time series to capture different aspects of the temporal changes in the time series and transform them into matrices so that they can be processed by 2DCNN-AE. For the LSTM-AE network, the matrices are taken and the rows are concatenated in a one-dimensional vector. As in [22] reconstruction error is used for the identification of outliers. A variant of this work is presented by Kieu et al. [27] As the main difference, they propose the use of sparse variants of recurrent networks grouped into ensembles to reduce overtraining in outlier detection, leading them to outperform the compared models.

In this work, we propose a method based on stacked autoencoder neural networks for the identification of anomalous values in the trajectory of vehicles measured by GPS. These neural networks do not require labels, so they can be adapted to this problem. To evaluate the performance of the method, we apply it to GPS measurements collected in the city of Santiago de Chile by a private company. The data set includes 13 million measurements considering 15,000 sensors. In summary, the contributions of this research can be summarized in

- Proposal of a stacked autoencoder neural network model for detecting anomalous sequences of GPS measurements in urban vehicles considering fully connected and convolutional architectures.
- Comparison of fully connected and convolutional autoencoder architectures.
- Evaluation of model results regarding anomalies detected by human users.

Our proposal mainly differs from the works by [20,22] since they use RNN and LSTM networks, while we propose fully connected and convolutional models additional to relative spatial relationships that can recognize outlier trajectories. Also, [21,23] models are based on autoregressive and neighborhood-based models that do not necessarily capture non-linear relationships between sequence measurements. The complex model presented in [25] considers maps with multiple trajectories, while we consider single trajectories. Unlike our proposal, the methods introduced in [26,27] consider aggregate features generated in a two-step process given by obtaining features based on time series differences and obtaining statistical features. Also, when performing the reconstruction of the time series, they consider the added features as new variables to be reconstructed, generating a 2D input matrix. In this way, the authors use typical operators of convolutional networks applied to images such as 2D convolution and max pooling. Instead, our proposed model is simpler since it considers 1D convolutions, which facilitate its implementation, reconstructing the original data given by the GPS measurement sequences. In general, the cited methods, including [26,27], are evaluated in labeled databases where the outliers correspond to the less frequent classes and in [25] outliers are artificially generated, while our proposed method is applied to a real unlabeled database during both training and testing.

## 2. Proposed Model for Detection of Outlier Trajectories

An alternative to detect outlier trajectories is by using classical outlier detection techniques. There are numerous alternatives to detect outliers such as models based on convex hull [28], clustering [29], k-nearest neighbours [30], among others. A classic density-based outlier detection model is given by LOF (Local Outlier Factor) [31]. This model is based on calculating the density of regions in the data using k-neighborhoods and declaring the objects in low-density regions as outlier values. The techniques mentioned above explore outlier values in their original data spaces and have been shown to work well on linearly separable distributions; however they tend to perform less well than nonlinear structures, according to [32]. An alternative to model non linear outlier patterns is by using autoencoder neuronal networks.

An autoencoder is an artificial neural network that tries to copy its input to its output, and internally compresses the input data into a hidden layer *h*, called *latent space*, with which it reconstructs the input data (see [33] for theoretical concepts and mathematical details). This data compression is based on reducing the number of neurons in the hidden layer, and, in this way, the network is forced to learn a representation of the main patterns of the input data. Since outliers often correspond to non-representative features, it is likely that the autoencoder network will not be able to reconstruct the outliers using the latent space, and this reconstruction allows us to flag as outliers those data that are not well explained using the latent space.

Autoencoders can be stacked to form a deep network. This neural network is called a stacked autoencoder and is typically made up of two interconnected subnets: the encoder and decoder networks. In general, the more layers there are in the encoder and decoder subnetworks, the more complex encodings this network will be able to learn.

**Encoder network:** It is formed by the input layer and a set of hidden layers: this subnet compresses the input data *x* to a latent space and the training of said network is done one layer at a time. Each layer is trained as an encoder by reducing the number of neurons and it receives as input the latent representation of the previous layer until reaching the hidden layer *h* that describes the latent space of the network. The encoder network is represented by the Equation (1).

(1) h=f(x)

**Decoder network:** It is formed by a set of hidden layers and the output layer: this subnet reconstructs the original input $\hat{x}$ based on the latent space *h* and the training of said network is done one layer at a time. Each layer is trained as a decoder by increasing the number of neurons and it receives as input the reconstruction of the previous layer until reaching the output layer, which has the number of neurons equal to that of the input layer. The network decoder is represented by the Equation (2).

(2) $$\hat{x}=g\left(h\right)$$

The stacked autoencoder can be represented by the Equation (3).

(3) $$g\left(f\left(x\right)\right)=\hat{x}$$

The implementation of autoencoder networks requires that they are not very complex because they can learn to memorize the input without obtaining a useful data representation [34]. In this work we implement the densely connected and convolutional neural networks for the encoder and decoder pair of the autoencoder network applied to the detection of outlier trajectories.

### 2.1. Densely Connected Stacked Autoencoder Network

**Figure 1:**
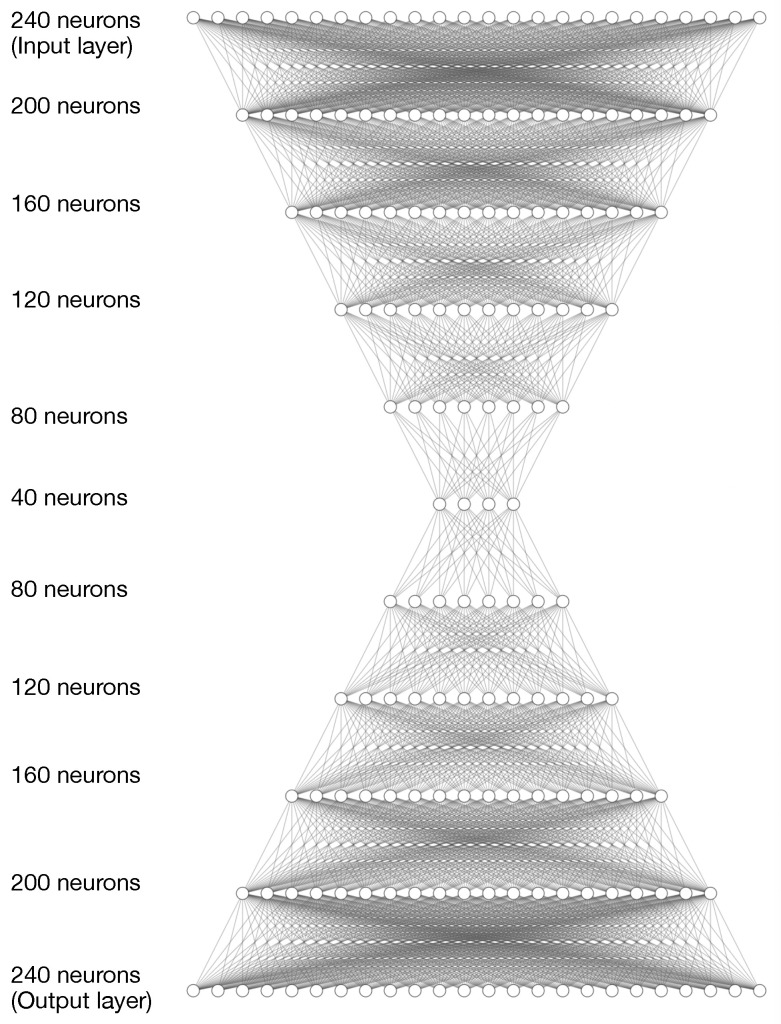
Example of a stacked autoencoder network architecture. This network considers five dense layers for both the encoder and decoder subnets made up of 240, 200, 160, 120, 80 and 40 neurons.

The first model uses densely connected layers, that is, all the neurons of a layer *L* are connected with all the neurons of the next layer L+1. Therefore the input of a neuron of the layer L+1 is the correlation of all the outputs of the neurons of layer *L*. The model receives as input a time series that has a set of multivariate observations and correlates all the observations which have six variables each, making the number of connections dependent on the number of observations and variables. To detect if a time series is an outlier, the model correlates the context of the time series, that is, all the variables of all the observations are associated. An example of an architecture used in experiments (Section 4) is shown in Figure 1. In this case, the neural network has five layers for the autoencoder and decoder subnetworks made up of 240, 200, 160, 120, 80 and 40 neurons, forming a typical symmetric structure in stacked autoencoder networks.

### 2.2. Stacked Convolutional Autoencoder Network

**Figure 2:**
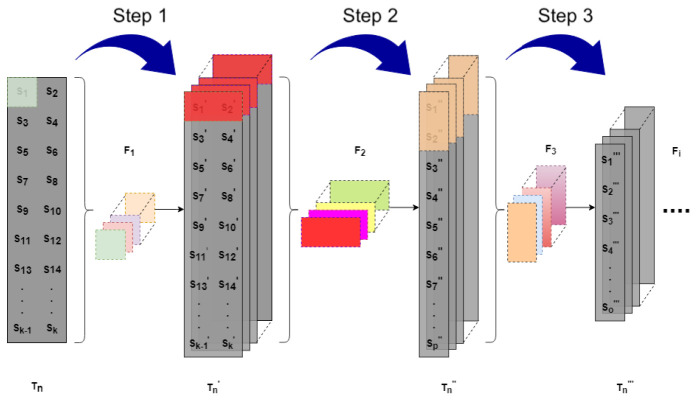
Proposed stacked convolutional neural network design.

The reason for using convolutional models is based on the idea that, if an observation has an anomalous behavior with respect to the adjacent observations, then it is not necessary to correlate all the observations. This second proposed model associates the set of neighboring observations, which reduces the number of connections. The model uses a filter or sliding window to correlate the neighboring observations, and for this it uses the convolutional layers that use filters that correlate the observations; this procedure is applied to the following layers. With this method, the model is not forced to look for correlations between all the observations; on the contrary, it allows us to correlate the observations that we want. Figure 2 shows the design of the stacked convolutional neural network.

This stacked convolutional autoencoder network receives as input a time series in the form of a two-dimensional grid. Next we will indicate the steps followed for the construction of the same in our proposal.

#### 2.2.1. Step 1

Given a time series $T_{n}=\left\{S_{1},S_{2},S_{3},\ldots ,S_{k}\right\}$, where $S_{i}=\left\{s_{i}^{1},s_{i}^{2},s_{i}^{3},\ldots ,s_{i}^{d}\right\}$, i=1,…,k, is a multidimensional observation $S_{i}\in R^{d}$, first the correlation between the observation variables $S_{i}$ is sought so that the size of the first filter is *d*, then the filter $F_{1}$ is applied to each observation obtaining a transformed time series $T_{n}^{{}^{\prime }}=\left\{S_{1}^{{}^{\prime }},S_{2}^{{}^{\prime }},S_{3}^{{}^{\prime }},\ldots ,S_{k}^{{}^{\prime }}\right\}$, where $S_{i}^{{}^{\prime }}$ is the correlation of the variables of $S_{i}$ and is also one-dimensional, i.e., $S_{i}^{{}^{\prime }}\in R$.

#### 2.2.2. Step 2

Given the transformed time series $T_{n}^{{}^{\prime }}$, we look for the correlation for each row of the transformed series, which is made up of a pair of observations. Since $S_{i}^{{}^{\prime }}$ is one-dimensional, the size of the filter is two. The filter $F_{2}$ is applied to each row and a new transformed time series is obtained: $T_{n}^{{}^{\prime \prime }}=\left\{S_{1}^{{}^{\prime \prime }},S_{2}^{{}^{\prime \prime }},S_{3}^{{}^{\prime \prime }},\ldots ,S_{k}^{{}^{\prime \prime }}\right\}$, where $S_{i}^{{}^{\prime \prime }}$ is one-dimensional.

#### 2.2.3. Step 3

Unlike the previous steps where the size of the filters is always the same, in this step the correlation between observations with different filter sizes is sought. Specifically in this step the size of the filters and the displacements to move the filter can be defined. Then, given the transformed time series $T_{n}^{{}^{\prime \prime }}$, a filter $F_{3}$ is applied and a new transformed time series is obtained: $T_{n}^{{}^{\prime \prime \prime }}=\left\{S_{1}^{{}^{\prime \prime \prime }},S_{2}^{{}^{\prime \prime \prime }},S_{3}^{{}^{\prime \prime \prime }},\ldots ,S_{k}^{{}^{\prime \prime \prime }}\right\}$. This step is repeated until a latent space is obtained.

### 2.3. Models Implementation

For the two proposed models, the hyperbolic tangent activation function and stochastic gradient descent were used as the optimization algorithm. Regarding the evaluation metrics, the coefficient of determination and explained variance were used. Since it is a regression problem, the optimization function was the mean square error cost function. The demo source code of this work can be found at https://github.com/sagagk/Autoencoder_Outliers (accessed on 1 October 2022).

## 3. Preprocessing and Data Analysis

In this Section, we will first show the data preprocessing, and then a brief analysis of the available data.

### 3.1. Data Preprocessing

#### 3.1.1. Data Description

Data for this experiment were provided by Waypoint Telecomunicaciones S.A. company. Specifically, the route history between 2016 and 2018 was considered and the data were exported in CSV files. The database contains approximately 30 million records in total (Given the privacy of the data, the access to them can be obtained on request by sending an e-mail to billy.peralta@unab.cl). Table 1 shows the description of each variable.

#### 3.1.2. Data Cleaning and Normalization

In this stage, the erroneous data records of the training set are corrected and cleaned, as well as the data are normalized to standardize the range of the model variables. The data provided by the company are complete and also do not contain erroneous data. Regarding data normalization, the min-max method was used.

#### 3.1.3. Transformation of Data into Time Series

**Figure 3:**
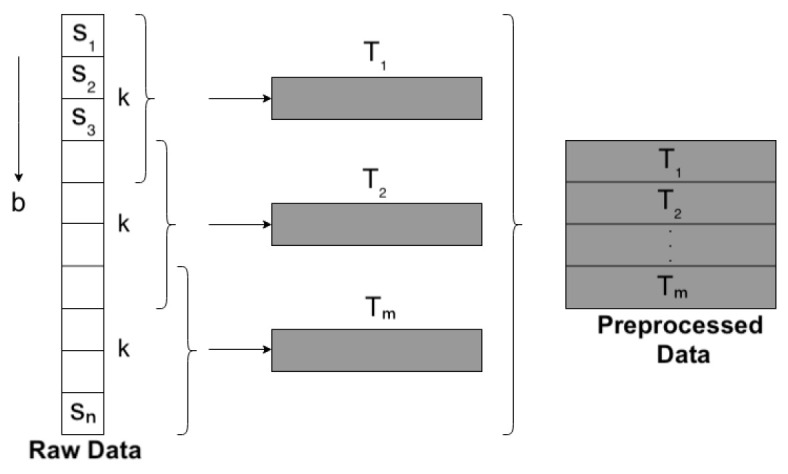
Data transformation to 1D representation.

The data extracted from the Waypoint database are vehicle records ordered in time. Each record is an observation at a time *t*, and different transformations are performed for the two proposed models since the data must be transformed into time series. Because the tested neural networks consider different input data formats, their preprocessing is shown below:

**(i).** The stacked autoencoder network receives multidimensional time series as input. The time series *T* is a sequence of observations $T=\{S_{1},S_{2},S_{3},\ldots ,S_{K}\}$, where $S_{i}=\left\{s_{i}^{1},s_{i}^{2},s_{i}^{3},\ldots ,s_{i}^{d}\right\}$ is a multidimensional observation $S_{i}\in R^{d}$, determining the size of the window *K* that will be considered with the set of observations. Note that the windows were obtained by sliding the same one in each sequence considering *b* steps; in this way the set of time series was obtained. In our case, we assigned *b* equal to 0.5K. The procedure is illustrated in Figure 3.

**Figure 4:**
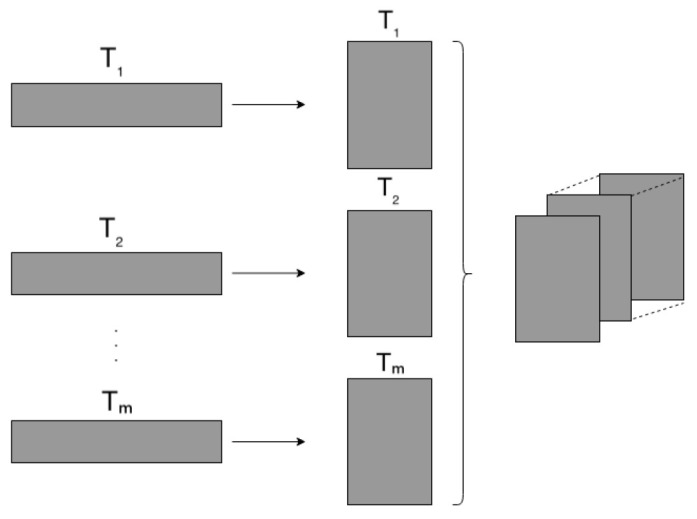
Data transformation to 2D representation.

**(ii).** The convolutional autoencoder network also receives time series as input but in a two-dimensional grid, as in the autoencoder network. We still use the sliding window, but now the time series *T* is transformed into a 2-dimensional grid; Figure 4 illustrates the procedure.

#### 3.1.4. Datasets

In order to test various configurations, four training sets were created. Since the objective is to detect outlier vehicle paths, the models must receive vehicle paths as input. In this paper we consider two sets of variables: the set of spatial variables (latitude, longitude and altitude), and the total set of six variables (which adds time, speed and nsat) represented in Table 1. In this way we propose to evaluate the effect of the selective use of spatial variables when detecting ourliers. We also cover two types of paths, short and long, to evaluate the effect of sequence length on performance (see, Table 2).

### 3.2. Exploratory Data Analysis

The objective of this brief study is to understand the general patterns of the data through basic statistics. To do this, we performed a descriptive analysis, represented in Table 3. Note that this table considers the original values of the available data, which is why obviously wrong extreme values appear. We propose to minimize the use of data preprocessing in order to test the models proposed in the detection of any type of outliers.

The univariate analysis shows that the *latitude* variable reaches a maximum value of 22.44, which does not coincide with the geographical location of the vehicles that transit in Chile and, therefore, is a possible reading error of the GPS device. The same problem occurs with the *longitude* variable and its maximum value of 0 which represents the location of Africa. Regarding the altitude variable, a very erroneous GPS reading value (3.57 × 10${}^{14}$) was eliminated from the analysis because it significantly altered the statistics of this variable. Then, the minimum and maximum values of this variable turned out to be −1044 and 5278, respectively, which indicate the presence of erroneous GPS values since most of the monitored vehicles were transiting at altitudes up to 650 m above the sea level.

The *speed* variable shows a negative value −9, which is a reading error since the speed is a positive value, while the number of satellites has a maximum value of 315, which indicates an erroneous GPS reading and is also an outlier value if compared to its median.

## 4. Experiments

For the experiments, the Hold-Out method was used to train and evaluate the model, considering 70% for training and 30% for testing. Since this problem corresponds to a regression, the mean square error was used as the cost function. The mean square error (MSE) given by

(4) $$MSE=\frac{1}{n}\sum_{i=1}^{n}{\left({\hat{x}}_{i}-x_{i}\right)}^{2}$$

provides an average error between the difference of the original data and reconstructed data, where *x* is the time series of the variables GPS, given in Table 1, $\hat{x}$ is the time series of GPS variables reconstructed by the model and *n* is the number of observations. The GPS variables correspond to the measurements obtained by the GPS sensor in the trajectory of a vehicle.

For the quantitative evaluation, the validation metrics used in this project are the coefficient of determination ($R^{2}$) and the explained variance (EV). The coefficient of determination measures the goodness of the fit made by the model and allows deciding whether the linear fit is sufficient, while the explained variance measures the proportion to which a model explains the variation (dispersion) of a given dataset. In the case of the subjective evaluation, the opinion of an expert was considered to establish whether a time series is an outlier or not based on the reconstruction error. Therefore, those time series with greater reconstruction error are labeled as outliers, while those with less reconstruction are considered as normal. In this case, we opted to consider the 100 time series with the highest reconstruction error as outliers. This is a number that experimentally appears reasonable in this dataset since the expected anomalies appear; on the other hand, with this method we avoid looking for a threshold which is not trivial to find. The experts in the field will evaluate each time series, giving an assessment of whether the time series is an outlier, a dubious one or not an outlier. The assessment used is shown in Table 4. The scale that appears in this table is based on the subjective criteria of the human expert. The *Outlier* tag is given when one is absolutely sure that a path is outlier, *No outlier* if the opposite is the case, and *Dubious* in case one is not sure if the path is outlier.

### 4.1. Quantitative Results

An architecture of a neural network is the way the neurons inside it are organized. Three different architectures were trained for each dataset, maintaining the mentioned configurations but varying the number of layers and number of neurons for each architecture. All architectures were trained with the same number of epochs (300) to maintain the same learning conditions. To specify each architecture, we will use the following notation that denotes the layer types that were used:

- D = Dense or fully connected layers
- C = Convolutional layers
- TC = Transposed or deconvolutional layers

In the experiments for the four databases, the three best results are shown for each database considering the error in the training set in both the full-connected and convolutional autoencoders. The former are denoted by AFC-A, AFC-B and AFC-C, while the latter by ACONV-A, ACONV-B and ACONV-C. The detail of the main architectures is shown in the text that accompanies each table of results.

**Figure 5:**
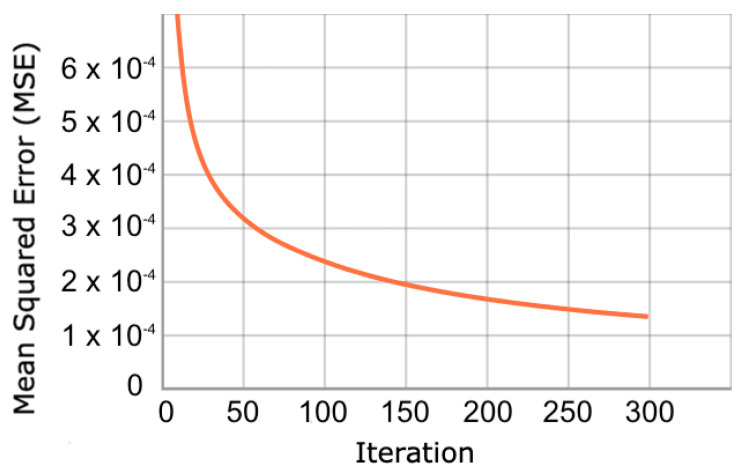
Evolution of training cost function. The function decreases by minimizing the reconstruction error of the trajectories in the training set.

**Figure 6:**
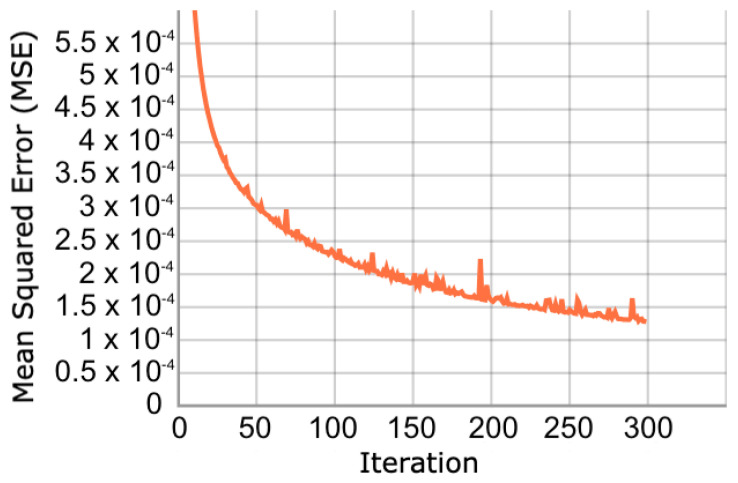
Evolution of validation cost function. The function decreases by minimizing the reconstruction error of the trajectories in the validation set.

In relation to the convergence of the optimization process, we show the evolution of the cost function in dataset 4. Figure 5 shows that the cost function during training converges gradually until iteration 300. When reviewing the validation set, the same behaviour is observed (Figure 6). This behavior is expected because the validation and training samples come from the same dataset, and the vast majority of GPS trajectories are not outliers. In the other databases the convergence patterns appear similar.

The pattern of convergence is repeated in the other datasets, and the same is true when measuring the adjusted $R^{2}$ metric. Next we will indicate the results obtained in all the datasets.

#### 4.1.1. Dataset 1

Table 5 shows the results of the autoencoders in dataset 1. In relation to full-connected architecture, it is observed that architecture C obtains the best result, which has 6D while architecture A and architecture B have 4D. In relation to convolutional models, it is observed that architecture B obtained the best result, which has 2C, 2D and 2TC; architecture A has 3C, 2D and 3TC and architecture C has 2C, 2D and 2TC. Architectures B and C have a smaller filter than architecture C. In comparative terms, it is observed that the smallest reconstruction error is reached in the convolutional variant.

#### 4.1.2. Dataset 2

Table 6 shows the results of the autoencoders in dataset 2. Regarding the full-connected architecture, note that architecture B obtained the best result, which has 8D; architecture A and C have 6D. In relation to convolutional models, it is observed that architecture A obtained the best result, which has 4C, 2D and 4TC; architecture B has 3C, 2D and 3TC and architecture C has 4C, 2D and 4TC. The filter of architecture A is smaller than that of the other architectures.

#### 4.1.3. Dataset 3

Table 7 shows the results of the autoencoders in dataset 3. In relation to the full-connected architecture, observe that architecture B obtained the best result, which has 4D; architecture A has 6D and architecture C has 4D. In relation to convolutional models, it is observed that architecture B obtained the best result, which has 3C, 2D and 3TC; architecture A has 3C, 2D and 3TC and architecture C has 2C, 2D and 2TC. The filter of architecture B is smaller than that of architectures A and C.

#### 4.1.4. Dataset 4

Table 8 shows the results of the autoencoders in dataset 4. In relation to full-connected architecture, it is observed that architecture C obtained the best result, which has 10D; architecture A has 8D and architecture B has 6D. In relation to convolutional models, it is observed that architecture A obtained the best result, which has 4C, 2D and 4TC; architecture B has 4C, 2D and 4TC and architecture C has 3C, 2D and 3TC. The filter of architecture A is smaller than that of architectures B and C.

### 4.2. Qualitative Network Results

The qualitative evaluation was only applied to the best architecture of the stacked autoencoder and stacked convolutional autoencoder of each dataset. The results show that the worst qualitative result was obtained from the convolutional stacked autoencoder applied to dataset 2. Analyzing the results of the autoencoder, it was observed that the vast majority of the paths are focused on a specific region of the map. It is very likely that the autoencoder found specific geo-location patterns in that region with respect to other regions. Since this is an unsupervised learning problem, it is not known what the result will be for the proposed models. In the four datasets, abrupt changes in speed between consecutive observations greater than 100 km/h were found, which were taken as anomalous observations. The proposed models also found the spatial outliers in the four datasets. Sudden speed changes and spatial outliers were counted as detected outliers. In Table 9 the mean and standard deviation of the percentage of detected outliers when applying a sampling techniques based on bootstrapping [35] is reported. In particular, we considered 1000 random samples with replacement from the list of the top 100 candidates according to each algorithm. The top candidates are obtained from the test set and are based on the given ranking by maximizing the reconstruction error. Each candidate is labeled by the expert according to Table 4. The results indicate that the most effective method is the stacked convolutional autoencoder when applied to dataset 4, obtaining an average of 82.1% accuracy, while the second most effective method is the standard stacked autoencoder with 10 layers considering the same dataset with an average of 79.5 %. On the other hand, the worst results appear in Dataset 2 where the convolutional model reaches a performance of 4% on average.

#### 4.2.1. Results of the Time Series Visualized on the Map

This section shows two visual examples of outlier time series detected by the model and two examples of time series that are not outliers but that the model detected as outliers with their respective maps and values. These visual examples are extracted from the 100 time series with the highest reconstruction error. In this case, the best neural network from each dataset that was previously evaluated is considered. Due to space optimization, we will only show examples about dataset 4. The examples show maps in Google Maps, where vehicle trajectories are marked by sequentially ordered light blue points, while the measurements that contribute the greatest error according to the neural network are marked by red dots. The *anomalous GPS measurements within trajectories* are obtained by considering those measurements that provide more than 90% reconstruction error in an anomalous trajectory according to the stacked autoencoder. The reason for flagging these measurements is to better understand the reason for anomalous labeling according to the neural network.

##### Examples of Correctly Detected Outlier Paths

**Figure 7:**
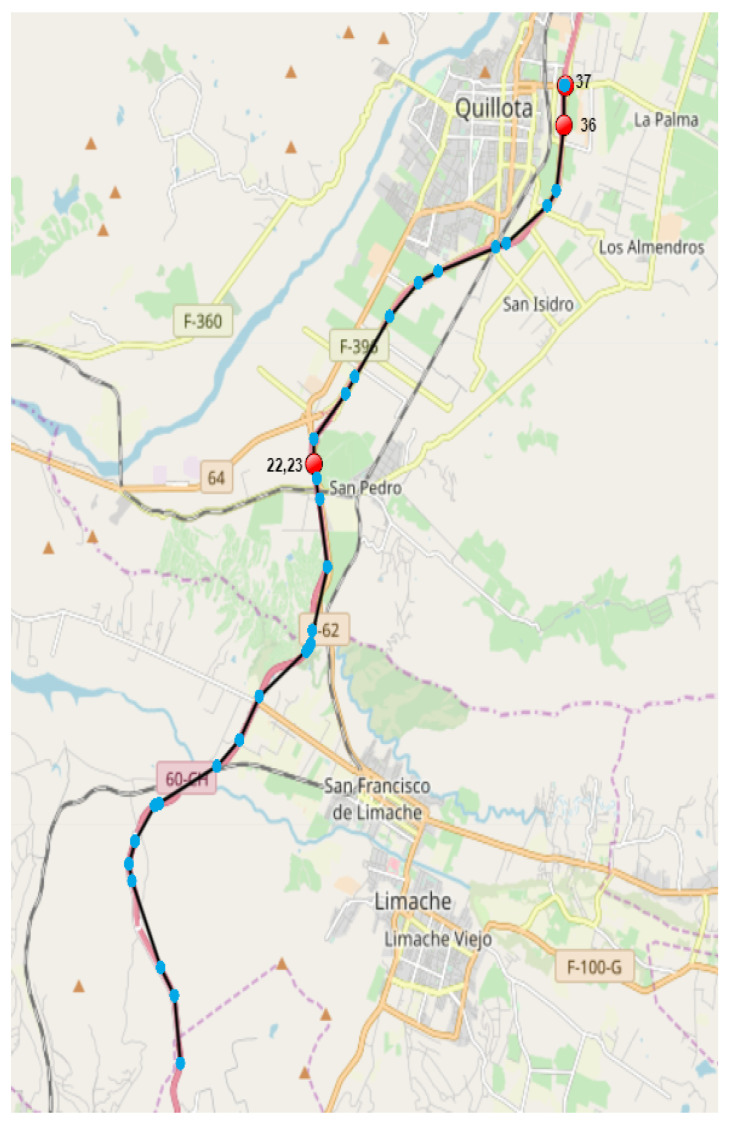
Example 1 of correctly detected outlier trajectory. The light blue points indicate GPS measurements of the complete trajectory, while the red points indicate the points with the highest reconstruction error according to the neural network.

Figure 7 shows a trajectory of 37 GPS measurements on a road, indicated by light blue points ordered from bottom to top, which generally presents normal continuity, except for measurements 22 and 23 that show a speed of 0, indicated by red dots which correspond to abrupt changes with respect to close measurements. In particular, the previous measurement, 21, had a speed of 95 km/h 40 s earlier, while the next measurement, 24, had a speed of 107 km/h 31 s later. On the other hand, observations 36 and 37, indicated by the red dots, show speeds of 151 km/h and 33 km/h in 30 s, which correspond to an unusual speed change according to the network. We indicate that this detection is correct, since the human expert agreed that there were anomalies in this trajectory according to the explanations given at the beginning of this paragraph. For greater clarity, Table 10 shows the values of the GPS measurements with the greatest error of this anomalous trajectory of Figure 7 together with the contiguous measurements.

**Figure 8:**
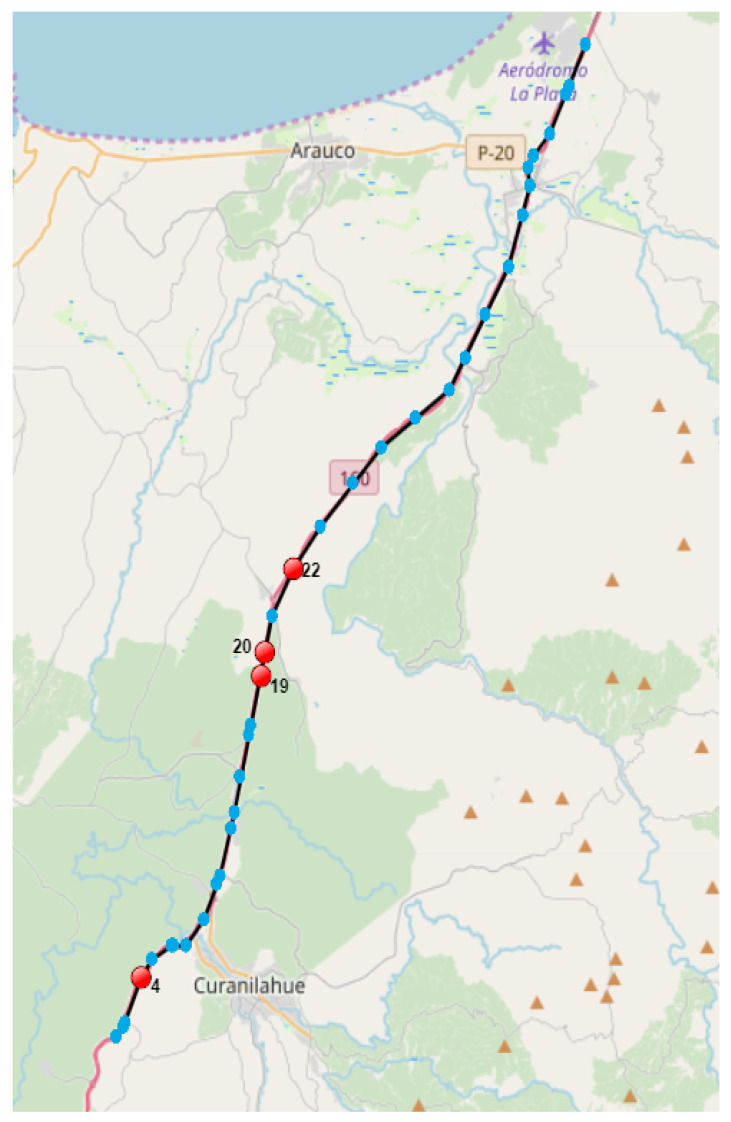
Example 2 of correctly detected outlier trajectory. The light blue points indicate GPS measurements of the complete trajectory, while the red points indicate the points with the highest reconstruction error according to the neural network. See detail in text.

The trajectory measurements shown in Figure 8, taken on a rural highway, still appear to have a mostly typical behavior. However, in measurement 4 there is a speed increase of 74 km/h in 40 s compared to the previous measurement, reaching 161 km/h. In measurements 19 and 20, it is observed that the speed decreases from 150 to 5 km/h in 40 s. On the other hand, in measurement 22 a speed of 167 km/h is obtained when the speeds of the previous measurements were 5 and 143 km/h at 80 and 40 s before. The human expert found these speed changes at such times unusual, even when they correspond to rural roads, which is why it is an example of an outlier trajectory. For greater detail, Table 11 shows the values of the GPS measurements with the greatest error of this anomalous trajectory of Figure 8, again considering the measurements adjacent to those with the greatest error.

##### Example of Normal Paths Detected as Outliers

**Figure 9:**
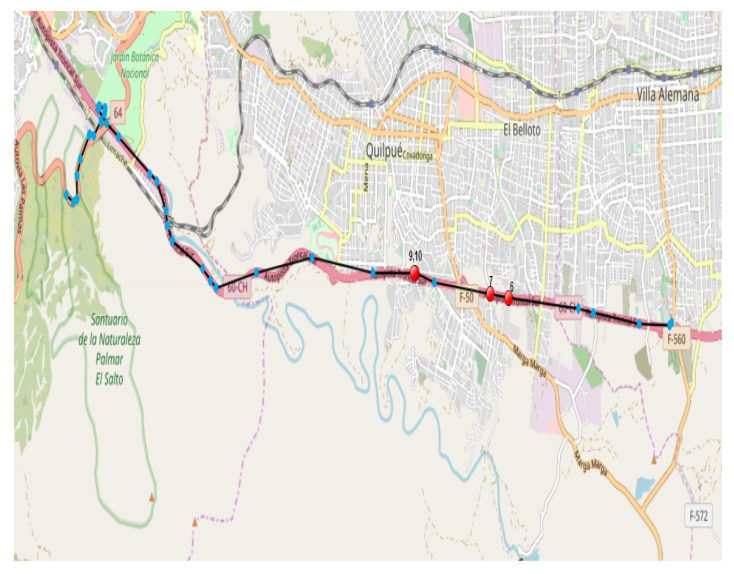
Example of a normal trajectory according to human expert detected as outlier by neural network. The light blue points indicate GPS measurements of the complete trajectory, while the red points indicate the points with the highest reconstruction error according to the neural network. See detail in text.

The trajectory measurements shown in Figure 9 have a typical behavior according to experts. This trajectory corresponds to a vehicle on the road. When examining the measurements with the greatest reconstruction error, it appears that in measurements 6 and 7 there are no large changes in velocity or displacement. On the other hand, in measurements 9 and 10, a noticeable speed change appears. However, when analyzing the area, the expert indicated that it is a normal event in his opinion due to car braking and subsequent acceleration. In this case, we propose that the neural network detects abrupt changes in speed as outliers. For greater detail, Table 12 shows the values of the GPS measurements with the greatest path error in Figure 8, once again considering the measurements adjacent to those with the greatest error.

### 4.3. LOF Classical Model

To complement our study, we considered the Local Outlier Factor (LOF), a classic outlier model. For this method only dataset 4 was used, i.e., the one which contains the velocity outliers. The LOF method receives the time series as input and returns a vector with values 1 and −1, where the value 1 indicates that the time series is an outlier and the value −1 indicates that the time series is normal. Each observation has six variables: date (*f*), latitude (lat), longitude (lon), altitude (alt), speed (*v*), and number of satellites (sat). For the detection of outlier observations, linear regressions were used, and each variable was taken, one at the time, as a dependent variable depending on the other ones, treated as explanatory variables. Furthermore, every dependent variable was taken as dependent also on its value at the previous time. We applied this method for each variable, and six linear regressions were obtained in the following way.

$$\begin{array}{c} f_{t}=F\left(f_{t-1},lat_{t},lon_{t},alt_{t},v_{t},sat_{t}\right)\\ lat_{t}=F\left(f_{t},lat_{t-1},lon_{t},alt_{t},v_{t},sat_{t}\right)\\ lon_{t}=F\left(f_{t},lat_{t},lon_{t-1},alt_{t},v_{t},sat_{t}\right)\\ alt_{t}=F\left(f_{t},lat_{t},lon_{t},alt_{t-1},v_{t},sat_{t}\right)\\ v_{t}=F\left(f_{t},lat_{t},lon_{t},alt_{t},v_{t-1},sat_{t}\right)\\ sat_{t}=F\left(f_{t},lat_{t},lon_{t},alt_{t},v_{t},sat_{t-1}\right). \end{array}$$

The calculated linear regressions were then applied to the observations of the time series, obtaining a prediction of the time series. To decide which observation was an outlier, the reconstruction error criterion was used. For the evaluation of the model, we only used the qualitative evaluation because this algorithm has a lazy learning, that is, it does not have a learning process.

The LOF was implemented in Python using the Scikit-Learn library [36]. Within the LOF configuration the default parameters were used [36], except for the number of neighbours, where 20 close neighbours were considered. In the qualitative evaluation of the LOF, 0% of outlier time series were detected: this result is due to the fact that the LOF does not detect outliers in sequential data but only in independent instances. Furthermore, it does not relate the variables as a neural network does, but it detects outliers based on the density of the data.

### 4.4. Explainable Model

Finally, it is known that deep neural models lack simple means of explanation. In order to understand the decisions of the neural network, a decision tree is used, which generates a decision rule that helps us understand the decisions of the neural network. As the decision tree has a supervised learning, we need the labeling of the training and validation data; for this we will use the given threshold based on a percentage of 99%. That is, the time series greater than the threshold are labeled as outliers, while the time series less than the threshold are labeled as normal.

The decision tree was implemented in Python 3.7 using the Scikit-Learn library. Within the decision tree configuration the default parameters were used, except the maximum depth which was taken equal to 20 levels. The decision tree obtained a precision of 92%.

The input of the decision tree is a time series, which is formed by 40 observations. To denote the observations and the variables of each observation, the following expression will be used: date *x*, lat_*x*, lon_*x*, alt_*x*, vel_*x*, nsat_*x*, where lat is the latitude variable, lon is the longitude variable, alt is the altitude variable, vel is the velocity variable, and nsat is the number of satellites; and *x* denotes the observation number that ranges from 1 to 40. To explain the decision tree, one pure sheet with an outlier time series decision and another pure sheet with a normal time series decision were taken.

**Outlier example:**Table 13 shows the decision rules, where the speeds of observations 6 and 8 are on average less than or equal to 41.5 km/h, unlike the speeds of observations 13 and 15, which are on average greater than or equal to 79.5 km/h, which indicate a sudden change in speed. Also the speeds of observations 17, 19, 21, 26, 29 and 31 are on average less than or equal to 29 km/h and show a sudden change in speed with respect to the speeds of observations 13 and 15.

**No outlier example:**Table 14 shows the decision rules, where the speeds of observations 1 to 19 are on average less than or equal to 45 km/h, which corresponds to a normal average speed with which the vehicles travel.

## 5. Discussion

Initially the Waypoint company identified two types of outliers in data from multivariable GPS sensors, which we call spatial outlier and speed outlier, where the first it is associated with sudden changes in GPS position, while the second is associated with sudden changes in speed. Four datasets were created where the objective of dataset 1 and dataset 2 was to detect spatial outliers in short and long paths, respectively, using the two proposed models. However, when applying dataset 2 with long paths to the stacked convolutional autoencoder network, we obtained a performance of 5% and the vast majority of paths were focused on a specific area of the map. On the other hand, the objective of dataset 3 and dataset 4 was to detect all types of outliers, including speed outliers in short and long distances respectively using the proposed models. In both datasets it was possible to detect the spatial outlier, but also the models detected abrupt or sudden changes in speed in the paths and were labeled as outliers. When analyzing the results, we observe that the outliers in short routes are better detected considering spatial variables, while the outliers in long routes are better detected considering all the variables. However, when comparing both types of routes, in general the outliers appear better detected when considering the long routes with all the available variables applying a stacked convolutional autoencoder model with an approximate improvement of 30% compared to the best model that considers only spatial variables.

Analyzing these sudden changes in speed in more detail, it was determined that this anomalous behavior may have a similarity with the abnormal behaviors of drivers. For example, when drivers encounter dangerous locations on the road and use the brakes hard to slow down or when brake due to some unforeseen event on the road that forces them to brake abruptly, such maneuvers can be called anomalous behavior of drivers and appear in the time series detected as outliers.

One aspect to improve is the interpretability of the neural model. In this work we consider the use of the decision tree to better understand the results obtained, however this model considers a univariate scheme when making a decision in each of the tree, which may be suboptimal. An important proposal to explore is the use of interpretability agnostic models of neural networks such as LIME [37] and SHAP [38] algorithms.

## 6. Conclusions and Future Work

This work presents a proposal for outlier data detection applied to vehicle trajectory. This tasks consists on to separate the observations with normal behavior from some observations that have an anomalous behavior; typically in these problems this anomalous behavior is known but those data that are outliers are not identified. Therefore, we are facing an unsupervised learning problem because we have not access to labelled outliers.

Our proposal uses deep autoencoders with full-connected and convolutional neural networks. The best model given by a stacked convolutional autoencoder detects on average 82.1% of outliers detected by human expert which indicate that this method is promising. The results indicates that the convolutional variants are more reliable to reconstruct long trajectories. In fact, they obtain better results than full-connected variants when considering long paths and all variables. We think that this behaviour is explained by the greater adaptability of CNNs to represent spatial patterns in long paths using all variables. On the other hand, the lower performance in the other configurations suggests that the fully connected autoencoder model is a better alternative when having short routes or only spatial variable information from GPSs. Note that in these cases the performance is 29.4% lower compared to the best global model.

As future work, it is planned to extend dataset 4 by adding new variables such as acceleration and direction of the vehicle, which can potentially improve the capacity of the models and enrich the detection of anomalous behavior of drivers. For example, by identifying locations of routes where drivers frequently use the brakes hard or make sudden changes of direction this can mean dangerous roads and therefore it is important to identify them, as they can help improve the design and surface of the roads and help prevent accidents. The detection of anomalous behavior of drivers opens many application areas such as recommendation of personalized routes warning of dangerous roads, in the monitoring of vehicles detecting interruptions in the journey which go against the road safety policy of a certain company, in the improvement of road design etc., and all these applications can be centralized in an intelligent transport system.

## Figures and Tables

**Table 1:** Description of variables.

Variable	Description
Time	UTC date and time of GPS transmission in milliseconds (ms).
Latitude	Decimal coordinates for GPS latitude.
Longitude	Decimal coordinates for GPS longitude.
Altitude	Altitude above the sea level surface.
Speed	Speed calculated by GPS equipment in Km/h.
Nsat	Number of satellites viewed by the GPS equipment.

**Table 2:** Summary of datasets used in experiments.

Dataset	Input Data Type	Trajectory Type
Dataset 1	Spatial variables	Short trajectories
Dataset 2	Spatial variables	Long trajectories
Dataset 3	Total variables	Short trajectories
Dataset 4	Total variables	Long trajectories

**Table 3:** Table of quantitative description of variables. Note that we consider the original data, which is why the measurement errors of GPS devices are included.

Variable	Type	Minimum	Maximum	Mean	Median
Latitude	Continuous	−53.67	22.44	−33.40	−33.59
Longitude	Continuous	−75.75	0	−71.36	−71.18
Altitude	Continuous	−1044	5278	445.45	207
Speed	Continuous	−9	201	11.02	0
Number of satellites	Discrete	0	315	9.88	9

**Table 4:** Evaluation table for an expert.

Evaluation	Degree
Outlier	1
Dubious	0.5
No outlier	0

**Table 5:** Stacked autoencoder results for the dataset 1. The boldface indicates the best results.

	MSE		$R^{2}$	
	Train	Test	Train	Test
AFC-A	3.888 × 10${}^{-7}$	1.3088 × 10${}^{-7}$	0.99997	0.999985
AFC-B	3.6265 × 10${}^{-7}$	1.234 × 10${}^{-7}$	0.99997	0.999987
**AFC-C**	**2.9139 × 10${}^{-7}$**	**1.0942 × 10${}^{-7}$**	**0.99998**	**0.999987**
ACONV-A	2.7117 × 10${}^{-7}$	1.0446 × 10${}^{-7}$	0.999983	0.9999879
**ACONV-B**	**2.4559 × 10${}^{-7}$**	**9.1347 × 10${}^{-8}$**	**0.999985**	**0.9999897**
ACONV-C	2.5914 × 10${}^{-7}$	9.4499 × 10${}^{-8}$	0.999984	0.9999894

**Table 6:** Stacked autoencoder results for the dataset 2. The boldface indicates the best results.

	MSE		$R^{2}$	
	Train	Test	Train	Test
AFC-A	1.335 × 10${}^{-6}$	8.3177 × 10${}^{-7}$	0.999915	0.99990
**AFC-B**	**8.633 × 10${}^{-7}$**	**4.099 × 10${}^{-7}$**	**0.999945**	**0.99995**
AFC-C	8.8594 × 10${}^{-7}$	4.56 × 10${}^{-7}$	0.999943	0.99994
**ACONV-A**	**1.7698 × 10${}^{-6}$**	**1.3554 × 10${}^{-6}$**	**0.9999**	**0.9998**
ACONV-B	2.9682 × 10${}^{-6}$	2.3015 × 10${}^{-6}$	0.9998	0.9997
ACONV-C	5.4754 × 10${}^{-6}$	4.3883 × 10${}^{-6}$	0.9997	0.9995

**Table 7:** Stacked autoencoder results for the dataset 3. The boldface indicates the best results.

	MSE		$R^{2}$	
	Train	Test	Train	Test
AFC-A	1.2654 × 10${}^{-4}$	1.1692 × 10${}^{-4}$	0.9976	0.9959
**AFC-B**	**4.9402 × 10${}^{-6}$**	**4.2375 × 10${}^{-6}$**	**0.9999**	**0.9999**
AFC-C	8.8641 × 10${}^{-5}$	7.4975 × 10${}^{-5}$	0.9983	0.9975
ACONV-A	1.6204 × 10${}^{-6}$	1.5368 × 10${}^{-6}$	0.99996	0.99995
**ACONV-B**	**1.2402 × 10${}^{-6}$**	**1.1958 × 10${}^{-6}$**	**0.99997**	**0.99996**
ACONV-C	1.2624 × 10${}^{-5}$	1.1092 × 10${}^{-5}$	0.99980	0.99960

**Table 8:** Stacked autoencoder results for the dataset 4. The boldface indicates the best results.

	MSE		$R^{2}$	
	Train	Test	Train	Test
AFC-A	2.255 × 10${}^{-4}$	2.1979 × 10${}^{-4}$	0.9957	0.9922
AFC-B	2.9738 × 10${}^{-4}$	2.8812 × 10${}^{-4}$	0.9943	0.9898
**AFC-C**	**1.3529 × 10${}^{-4}$**	**1.278 × 10${}^{-4}$**	**0.9974**	**0.9955**
**ACONV-A**	**1.0563 × 10${}^{-4}$**	**9.2084 × 10${}^{-5}$**	**0.9980**	**0.9980**
ACONV-B	8.2154 × 10${}^{-5}$	6.8964 × 10${}^{-5}$	0.9984	0.9976
ACONV-C	8.7996 × 10${}^{-5}$	7.5401 × 10${}^{-5}$	0.9983	0.9974

**Table 9:** Percentage of outliers detected considering the top 100 candidates from 1000 random samples: the mean and standard deviation (in parenthesis) are reported in the last column. The boldface indicates the best results.

Dataset	Neural Network	% Detected Outlier
Dataset 1	Stacked Autoencoder(6F)	*51.7 (5.0)*
	Stacked Convolutional Autoencoder(2C-2F-2D)	*39.1 (5.0)*
Dataset 2	Stacked Autoencoder (8F)	*46.8 (5.2)*
	Stacked Convolutional Autoencoder (4C-2F-4D)	*4.0 (2.0)*
Dataset 3	Stacked Autoencoder (4F)	*20.0 (3.7)*
	Stacked Convolutional Autoencoder (3C-2F-3D)	*15.0 (3.3)*
Dataset 4	Stacked Autoencoder (10D)	79.5 (4.1)
	Stacked Convolutional Autoencoder (4C-2F-4D)	**82.1 (3.8)**

**Table 10:** Detail of GPS measurements with the greatest error, including contiguous ones, of the trajectory of Figure 7. The measurements that most contribute to the trajectory error are indicated in boldface in Id.

Id	Date	Latitude	Longitude	Altitude	Speed
21	08:38:24	−32.937323	−71.287701	87.0	95.0
**22**	08:39:04	−32.934893	−71.288131	83.0	0.0
**23**	08:39:13	−32.934893	−71.288131	83.0	0.0
24	08:39:44	−32.931070	−71.288090	81.0	107.0
35	08:43:14	−32.893170	−71.233126	119.0	113.0
**36**	08:43:44	−32.883116	−71.231621	123.0	151.0
**37**	08:44:14	−32.876997	−71.231075	130.0	33.0
38	08:44:15	−32.876929	−71.231116	130.0	33.0

**Table 11:** Detail of GPS measurements with the greatest error, including contiguous ones, of the trajectory of Figure 8. The measurements that contribute the most to the trajectory error are indicated in boldface in Id.

Id	Date	Latitude	Longitude	Altitude	Speed
3	13:06:04	−37.486372	−73.393762	178.0	84.0
**4**	13:06:44	−37.473855	−73.387041	170.0	161.0
5	13:06:59	−37.469131	−73.383172	169.0	140.0
18	13:10:44	−37.404656	−73.345360	137.0	146.0
**19**	13:11:24	−37.390788	−73.341608	138.0	150.0
**20**	13:12:04	−37.384083	−73.339738	143.0	5.0
21	13:12:44	−37.374742	−73.337310	141.0	143.0
**22**	13:13:24	−37.361657	−73.329252	131.0	167.0
23	13:14:04	−37.349851	−73.318316	114.0	131.0

**Table 12:** Detail of GPS measurements with the greatest error, including contiguous ones, of the trajectory of Figure 9. The measurements that contribute the most to the trajectory error are indicated in boldface in Id.

Id	Date	Latitude	Longitude	Altitude	Speed
5	14:47:11	−33.066468	−71.394259	177.0	125.0
**6**	14:47:51	−33.065397	−71.411271	162.0	154.0
**7**	14:48:01	−33.064933	−71.415800	169.0	154.0
8	14:48:31	−33.063733	−71.429321	166.0	112.0
**9**	14:49:01	−33.062845	−71.433879	165.0	20.0
**10**	14:49:11	−33.062808	−71.434275	164.0	19.0
11	14:49:51	−33.062838	−71.444221	173.0	120.0

**Table 13:** Decision rule table for outlier time series.

Rule	Condition
1	vel_6 ≤ 44.5
2	vel_8 ≤ 38.5
3	vel_13 ≥ 78.5
4	vel_15 ≥ 80.5
5	vel_17 ≤ 26.5
6	vel_19 ≤ 13.5
7	vel_21 ≤ 34.5
8	vel_26 ≤ 32.5
9	vel_29 ≤ 23.5
10	vel_31 ≤ 43.5

**Table 14:** Decision rule table for normal time series.

Rule	Condition
1	vel_6 ≤ 44.5
2	vel_7 ≤ 42.5
3	vel_8 ≤ 38.5
4	vel_9 ≤ 43.5
5	vel_11 ≤ 72.5
6	vel_13 ≤ 43.5
7	vel_15 ≤ 36.5
8	vel_17 ≤ 26.5
9	vel_19 ≤ 13.5
10	vel_20 ≤ 26.5
11	vel_21 ≤ 34.5
12	vel_22 ≤ 42.5
13	vel_26 ≤ 32.5
14	vel_28 ≤ 43.5
15	vel_29 ≤ 23.5
16	vel_30 ≤ 42.5
17	vel_31 ≤ 43.5
18	vel_33 ≤ 53.5
19	vel_34 ≤ 47.5
20	lon_37 ≤ −64.114

## Data Availability

Not applicable.
